# Comprehensive Analysis of Non-coding RNA Profiles of Exosome-Like Vesicles From the Protoscoleces and Hydatid Cyst Fluid of *Echinococcus granulosus*

**DOI:** 10.3389/fcimb.2020.00316

**Published:** 2020-07-22

**Authors:** Xiaofan Zhang, Wenci Gong, Shengkui Cao, Jianhai Yin, Jing Zhang, Jianping Cao, Yujuan Shen

**Affiliations:** ^1^Chinese Center for Disease Control and Prevention, National Institute of Parasitic Diseases, Shanghai, China; ^2^Chinese Center for Tropical Diseases Research, Shanghai, China; ^3^Key Laboratory of Parasite and Vector Biology, Ministry of Health, Shanghai, China; ^4^WHO Collaborating Centre for Tropical Diseases, Shanghai, China; ^5^National Center for International Research on Tropical Diseases, Ministry of Science and Technology, Shanghai, China

**Keywords:** *Echinococcus granulosus*, exosome-like vesicles, miRNA, non-coding RNAs, parasite–host interactions

## Abstract

Cystic echinococcosis is a worldwide chronic zoonotic disease that threatens human health and animal husbandry. Exosome-like vesicles (ELVs) have emerged recently as mediators in the parasite–parasite intercommunication and parasite–host interactions. Exosome-like vesicles from parasites can transfer non-coding RNAs (ncRNAs) into host cells to regulate their gene expression; however, the ncRNAs profiles of the ELVs from *Echinococcus granulosus* remain unknown. Here, we isolated protoscolece (PSC)–ELVs and hydatid fluid (HF)–ELVs from the culture medium for *E. granulosus* PSCs *in vitro* and the HF of fertile sheep cysts, respectively. The microRNA (miRNA), long non-coding RNA (lncRNA), and circular RNA (circRNA) profiles of the two types of ELVs were analyzed using high-throughput sequencing, and their functions were predicted using Gene Ontology enrichment and Kyoto Encyclopedia of Genes and Genomes pathway analysis. In PSC–ELVs and HF–ELVs, 118 and 58 miRNAs were identified, respectively, among which 53 miRNAs were present in both ELVs, whereas 65 and 5 miRNAs were unique to PSC–ELVs and HF–ELVs, respectively; 2,361 and 1,254 lncRNAs were identified in PSC–ELVs and HF–ELVs, respectively, among which 1,004 lncRNAs were present in both ELVs, whereas 1,357 and 250 lncRNAs were unique to PSC–ELVs and HF–ELVs, respectively. Intriguingly, the spilled PSCs from cysts excrete ELVs with higher numbers of and higher expression levels of miRNAs and circRNAs than HF–ELVs. The miRNA sequencing data were validated by quantitative reverse transcription–polymerase chain reaction. Furthermore, the target lncRNAs and mRNAs regulated by the 20 most abundant miRNAs were screened, and a ceRNA regulatory network containing 5 miRNAs, 41 lncRNAs, and 23 mRNAs was constructed, which provided new ideas and the molecular basis for further clarification of the function and mechanism of *E. granulosus* ELVs ncRNAs in the parasite–host interactions. Egr-miR-125-5p and egr-miR-10a-5p, sharing identical seed sites with host miRNAs, were predicted to mediate inflammatory response, collagen catabolic process, and mitogen-activated protein kinase cascade during parasite infections. In conclusion, for the first time, we identified the ncRNAs profiles in PSC–ELVs and HF–ELVs that might be involved in host immunity and pathogenesis, and enriched the ncRNAs data of *E. granulosus*. These results provided valuable resources for further analysis of the regulatory potential of ncRNAs, especially miRNAs, in both types of ELVs at the parasite–host interface.

## Introduction

Cystic echinococcosis is a worldwide zoonotic disease that is caused by the metacestodes (larval stages) of the tapeworm *Echinococcus granulosus*. Humans may be infected by the accidental ingestion of *E. granulosus* eggs (McManus et al., 2012). *Echinococcus granulosus* infection is characterized by unilocular fluid-filled hydatid cysts within the internal organs (mainly the liver and lungs) of humans and other livestock intermediate hosts (Wen et al., 2019). It is endemic in countries and regions with extensive animal husbandry, causing huge economic losses and disease burden (Budke et al., 2006).

Hydatid cysts comprise cyst walls and cyst contents, such as the brood capsule, protoscoleces (PSCs), hydatid fluid (HF), and daughter cyst. The cyst walls consist of an inner germinal layer (GL) supported externally by a tough, elastic, acellular laminated layer, surrounded by a host-produced fibrous adventitial layer. The GL undergoes asexual multiplication to produce the brood capsule and large numbers of PSCs. In addition to its proliferative activity, the GL is involved in secretory activity, secreting several proteins into the HF that could play a role in host evasion (Monteiro et al., 2010). A proteomic analysis of the exosome-like vesicles (ELVs) from the HF of *E. granulosus–*infected fertile sheep identified a number of host proteins and parasite-derived proteins, among which antigen B and antigen five could reach the host and elicit a strong specific antibody response (Siles-Lucas et al., 2017). For *E. granulosus* PSCs, the excretory/secretory products can directly regulate the differentiation of B10, B17, and Th17 cells, and stimulated B10 cells produce interleukin (IL)10 via Toll-like receptor 2 (TLR2) signaling (Pan et al., 2017, 2018). A recent study isolated and analyzed the proteome of PSC–ELVs, which can be internalized by dendritic cells and induce their maturation (Nicolao et al., 2019). These studies mainly focused on the proteins in the two types of ELVs.

Extracellular vesicles (EVs) are small membranous vesicles that are generally classified in three major types, exosomes, microvesicles, and apoptotic bodies, based on their size, biogenesis, and composition. These EVs can carry a wealth of bioactive molecules such as proteins, carbohydrates, lipids, and nucleic acids, which are mainly found in specific vesicles known as exosomes. Exosomes are endocytic vesicles of ~30–150 nm in size that are released after the fusion of multivesicular bodies with the plasma membrane. Therefore, exosomes contain specific membrane markers such as ALIX, enolase, 14-3-3, CD63, and CD9 (Ying et al., 2017). Exosome-like vesicles have been identified in a number of parasite species, including several protozoa, trematodes, and nematodes, and act in intercellular communication and parasite–host interactions (Marcilla et al., 2014; Coakley et al., 2015). Exosome-like vesicles contain specific repertoires of non-coding RNAs (ncRNAs), including microRNAs (miRNAs), long non-coding RNAs (lncRNAs), and circular RNAs (circRNAs), which regulate gene expression at the levels of transcription, RNA processing, and translation (Cech and Steitz, 2014; Lambertz et al., 2015; Fan et al., 2018). MicroRNAs are small single-stranded ncRNAs molecules with a length of ~22 nt. As key posttranscriptional regulators, miRNAs are capable of regulating gene expression by degrading or suppressing the target mRNA, mostly through specifically binding to the 3′ untranslated region (3′ UTR) (Shukla et al., 2011). Long ncRNAs are more than 200 nucleotides in length and have limited protein-coding potential, which are also involved in posttranscriptional regulation by interacting with miRNAs, mRNAs, or proteins (Guttman et al., 2013; Ulitsky and Bartel, 2013). Recent evidence has shown that parasite-derived ELVs ncRNAs are involved in the regulation of host gene expression upon internalization of ELVs (Garcia-Silva et al., 2014a) and have been implicated in the host immune response and pathogenesis of a variety of parasite infections (Buck et al., 2014; Garcia-Silva et al., 2014b).

Helminth miRNAs encapsulated into ELVs and internalized by host immune cells induced regulation and expansion of regulatory T cells, resulting in the control of inflammation (Siles-Lucas et al., 2015). The miR-10 family and miR-277 were the most highly enriched miRNAs in ELVs of both *Dicrocoelium dendriticum* and *Fasciola hepatica* and seem to have potential immune-regulatory functions (Fromm et al., 2017). *Schistosoma japonicum*–derived ELVs contained miR-bantam, which may be involved in the hepatic pathogenesis of schistosomiasis (Zhu et al., 2016a). Parasite ELVs cargoes have been characterized mainly at the protein level, although other molecules, such as miRNAs, have been identified inside. However, there has been no study to identify and characterize miRNAs and other ncRNAs in *E. granulosus* PSC–ELVs and HF–ELVs.

In the present study, we showed that *E. granulosus* PSCs secrete EVs *in vitro*, and hydatid cysts produce EVs, whose size and morphology are consistent with exosomes. High-throughput sequencing was conducted to analyze the ncRNAs (miRNAs, circRNAs, and lncRNAs) profiles in the two types of ELVs, and bioinformatics analysis of the 20 most abundant miRNAs in the two types of ELVs was performed to explore the possible biological processes and pathways associated with pathogenicity and the host immune response. These ELVs contain small RNA species, including specific miRNAs that are homologous to host miRNAs with known immunomodulatory roles (Zhang et al., 2016; Vaher et al., 2019). The results demonstrated that the most abundant miRNAs in both types of ELVs might be new candidates for the mechanism of parasite–parasite communication and parasite–host interactions of ELVs in parasitic diseases.

## Materials and Methods

### Parasite Material

Liver hydatid cysts were collected aseptically from naturally infected sheep slaughtered at abattoirs located in Xinjiang Uygur Autonomous Region, China. The HF and PSCs were obtained from the fertile cysts under aseptic conditions and were placed in 50 mL falcon tubes. The HF was centrifuged at 2,000 × *g* for 5 min to remove the PSCs and other solid material. PSC-free HF (300 mL) from more than 10 fertile cysts was pooled and stored at −80°C until use. The PSCs were washed 5–8 times using 0.9% NaCl containing 100 U/mL penicillin and 100 μg/mL streptomycin (Invitrogen, Frederick, MD, USA) and maintained in sterile conditions until *in vitro* culture.

### *In vitro* Culture of PSCs

*In vitro* culture of PSCs (*n* = 20,000/75 cm^2^) and viability assays were carried out as previously described, with modifications (Cumino et al., 2010). Briefly, PSCs were cultured in RPMI (Roswell Park Memorial Institute) 1,640 medium (Gibco/Life Technologies, Carlsbad, CA, USA) supplemented with antibiotics (200 U/mL penicillin and 200 μg/mL streptomycin) and glucose (4 mg/mL) in 75 cm^2^ cell culture flasks at 37°C under 5% CO_2_. Viability was determined using a trypan blue dye exclusion test. The parasite culture medium was harvested and changed every 12 h. Briefly, 20,000 PSCs were maintained in serum-free media (300 mL) for 7 days. The medium collected from different time points was pooled and stored at −80°C until use.

### Purification of Exosome-Like Vesicles

Protoscolece–ELVs and HF–ELVs were isolated from the PSCs culture medium and the pooled HF, respectively, and enriched by differential centrifugation. The PSCs culture medium and pooled HF were centrifuged at 300 × *g* for 10 min, at 2,000 × *g* for 20 min, and finally at 10,000 × *g* for 40 min to remove large dead cells and cell debris. The supernatant was filtered using low-protein binding 0.22 μm pore filters (Millipore, Bedford, MA, USA). The filtered PSCs culture medium and HF were subjected to ultracentrifugation at 110,000 × *g* for 90 min at 4°C to pellet the vesicles using a Beckman Coulter Optima L-100 XP ultracentrifuge (Beckman Coulter, Indianapolis, IN, USA) with an SW 41 Ti rotor. The final pellets were washed with phosphate-buffered saline (PBS) and subjected to further ultracentrifugation at the same high speed. Protoscolece–ELVs and HF–ELVs were resuspended separately in 100 μL of PBS and stored at −80°C until use.

### Nanoparticle Tracking Analysis

Nanoparticle tracking analysis (NTA) was conducted using a NanoSight LM10 (NanoSight, Malvern Panalytical Ltd., Malvern, UK) to determine the size and frequency distribution of PSC–ELVs and HF–ELVs preparations, performed in triplicate. NanoSight analysis allows a better statistical resolution of vesicle size and concentration by measuring the Brownian motion of particles in solution related to particle size (Oosthuyzen et al., 2013).

### Transmission Electron Microscopy

Purified PSC–ELVs and HF–ELVs (both 10 μL) resuspended in 100 μL of PBS were added to 200-mesh formvar-coated grids (Agar Scientific Ltd., Stanstead, UK) for 1 min, and the remaining liquid was wiped off using filter paper. The grids were negatively stained with 3% phosphotungstic acid for 1 min and dried at room temperature. The grids were loaded onto the sample holder of the transmission electron microscope (TEM) (HITACHI, Tokyo, Japan) and exposed at 80 kV for image capture.

### Western Blotting

The ELV pellet (20 μL) was mixed with 10 μL of a solution of 5 × sodium dodecyl sulfate (SDS) and 20 μL of 1 × PBS and then boiled at 100°C for 10 min. Thirty micrograms of each protein sample was separated on 10% SDS–polyacrylamide gel electrophoresis, and the proteins were then transferred to polyvinylidene fluoride membranes (Millipore) at 15 W for 30 min. After a 1 h blocking step in Tris-buffered saline (TBS) 0.05% - Tween 20 (TBST) containing 5% bovine serum albumin, the membranes were incubated at 4°C overnight with the following primary antibodies: anti−14-3-3 zeta/delta (1/1,000), antienolase (1/1,000), and anti-CD9 (1/1,000) antibodies (Cell Signaling Technology, Danvers, MA, USA). For all secondary antibody incubations, horseradish peroxidase–conjugated or goat anti–rabbit antibodies (Cell Signaling Technology) were used at a 1:5,000 dilution. The membranes were visualized using an ECL Western blotting Detection System (Clinx Science Instruments Co., Ltd., Shanghai, China).

### Small RNA Library Construction, Sequence Analysis, and Identification of miRNAs

Total RNA containing small RNAs was extracted from PSC–ELVs and HF–ELVs using the mirVanaTM miRNA Isolation Kit (Ambion, Austin, TX, USA), following the manufacturer's protocol. The small RNA library construction and sequencing were conducted by OE Biotech Co., Ltd. (Shanghai, China). The quality, quantity, and integrity of the total RNAs were assessed using a NanoDrop 2,000 instrument (Thermo Fisher Scientific, Waltham, MA, USA), a Qubit 2.0 Fluorometer (Life Technologies), and an Agilent 2,100 Bioanalyzer (Agilent Technologies, Santa Clara, CA, USA), respectively. The small RNA libraries were constructed using TruSeq Small RNA Library Prep Kit (Illumina, San Diego, CA, USA) according to the manufacturer's instructions. Subsequently, RNA-Seq libraries were sequenced on the Illumina sequencing platform (HiSeq™ X Ten).

The basic reads were converted into raw data by base calling. Low-quality reads without a 3′ adapter and insert tag were filtered, and the reads with 5′ primer contaminants and poly (A) were removed. Clean reads with high quality and the specified length distribution (15–41 nt) in the reference genome were determined and then used for further analysis. These RNAs were aligned and then subjected to BLAST searching against the Rfam v.12.0 (Nawrocki et al., 2015) and GenBank databases. The ncRNA sequences annotated as rRNAs, tRNAs, small nuclear RNAs (snRNAs), and small nucleolar RNAs (snoRNAs) were removed. The remaining reads were matched against the miRBase v.21 database (http://www.mirbase.org/), and the known miRNAs were identified (Griffiths-Jones et al., 2008). The expression patterns of the known miRNAs in different samples were analyzed. Unannotated small RNAs were analyzed using mirdeep2 (Friedlander et al., 2012) to predict novel miRNAs. Based on the hairpin structure of a pre-miRNA and the miRBase database, the corresponding miRNA star sequences were also identified. The threshold set for upregulated or downregulated miRNAs was fold change ≥2.0 and *P* < 0.05.

### Quantitative Reverse Transcription–Polymerase Chain Reaction (qRT-PCR)

Total small RNAs from 100 μL of ELVs of both types were extracted using the miRNeasy Serum/Plasma Kit (Qiagen, Boston, MA, USA) according to the manufacturer's instructions. During the RNA purification step the same amount of cel-miR-39 (*Caenorhabditis elegans* miRNA; Qiagen) spike in control was added to each sample to monitor the efficiency of miRNA extraction and allowed for normalization of sample-to-sample variation. Three hundred nanograms of total RNA were used for reverse transcription. cDNA was synthesized using the miScript II RT Kit (Qiagen) according to the manufacturer's protocols. Quantitative reverse transcription–polymerase chain reaction (qRT-PCR) was performed to use a miScript SYBR Green PCR Kit (Qiagen) on a Bio-Rad CFX96 system. The primers used for the qRT-PCR are shown in Supplementary Table 1. The miRNA expression levels were quantified based on the threshold cycle (Ct) values. Cel-miR-39 served as the external control for the expression analysis of ELVs-derived miRNAs (Sohn et al., 2015; Lovett et al., 2018). The relative gene expression values for ELVs miRNAs were normalized to cel-miR-39 and calculated using the comparative Ct [2(–ΔΔCt)] method. Triplicate independent experiments were performed for each type of ELVs.

### Sequencing and Identification of lncRNAs and circRNAs

After extracting total RNA from the samples and removing the ribosomal RNA using a Ribo-Zero Gold rRNA Removal Kit (Illumina), the RNA was fragmented using fragment reagent. First-strand cDNA was synthesized using 8 μL of First Strand Synthesis Act D Mix and SuperScript II Reverse Transcriptase (Invitrogen) according to the manufacturer's protocols. Second-strand cDNA was synthesized using 5 μL of End Repair Control and 20 μL of Second Strand Marking Master Mix. The double-stranded cDNA was purified using AMPure XP beads (Beckman Coulter; cat. no. A63881). After repairing the ends and ligating the adenylated 3′ ends and sequence adapters, the DNA fragments were enriched via PCR. Finally, the products were purified (Agencourt AMPure XP; Beckman Coulter), and the library quality was assessed on an Agilent Bioanalyzer 2,100 system (Agilent Technologies). An Illumina sequencing platform (HiSeq™ X Ten) was used for sequencing, and 150-base-pair (bp) paired-end reads were generated.

Raw reads in the fastq format were subjected to quality preprocessing using Trimmomatic (0.36). After removing the adapter and low-quality reads, the clean reads were obtained. Hisat2 (2.2.1.0) was used to align the clean reads with the *E. granulosus* reference genome. For the mapped reads, transcripts were reconstructed based on the probability model and the comparison results of each sample using StringTie (1.3.3b). Finally, the candidate lncRNAs were identified using the software CPC (0.9-r2), CNCI (1.0), PFAM (v30), and PLEK (1.2), which were used to predict the coding capacity of the transcripts. The clean reads were analyzed by CIRI software for circRNAs prediction. The abundance of lncRNAs and circRNAs was calculated using FPKM (fragments per kilobase of transcript per million mapped reads), and RPM (reads per million reads), respectively. The differentially expressed lncRNAs and circRNAs were detected using a negative binomial distribution test based on the DESeq package (1.18.0). The threshold set to identify differentially expressed lncRNAs and circRNAs was fold change ≥2.0 and *P* < 0.05.

### Target Prediction and Functional Annotation of the 20 Most Abundant miRNAs

The candidate host target genes of the 20 most abundant miRNAs in PSC–ELVs were predicted using the software miRanda v3.3a (Betel et al., 2010) with the following parameters: S ≥ 150; ΔG ≤ −30 kcal/mol; and demanding strict 5′ seed pairing. To analyze the biological functions of ELVs miRNAs, the predicted miRNA target genes were annotated using Gene Ontology (GO) enrichment and the Kyoto Encyclopedia of Genes and Genomes (KEGG) pathway analysis. Gene Ontology analysis labels these target genes with a function, such as biological process, molecular function, or cellular component. Kyoto Encyclopedia of Genes and Genomes analysis provides the information of signal transduction and disease pathways for target genes, thus providing a basis for research into the function and involved pathways of ELVs miRNAs. The GO enrichment and KEGG pathway enrichment analysis of the 20 most abundant miRNA-target genes were both performed using R software (v3.6; https://www.r-project.org/) based on the hypergeometric distribution.

### Construction of the ceRNA Regulatory Network

Regulatory relationships between ELVs miRNAs and host mRNAs and between ELVs miRNAs and lncRNAs were predicted by Shanghai OE biotech Co., Ltd., using miRanda v3.3a. Based on the ELVs miRNA–mRNA interaction analysis and the miRNA–ELVs lncRNA interaction analysis, an lncRNA–miRNA–mRNA regulatory network was constructed and visualized using Cytoscape (v3.6.1; https://cytoscape.org) software.

## Results

### Identification of Exosome-Like Vesicles From HF and PSCs

**Figure 1:**
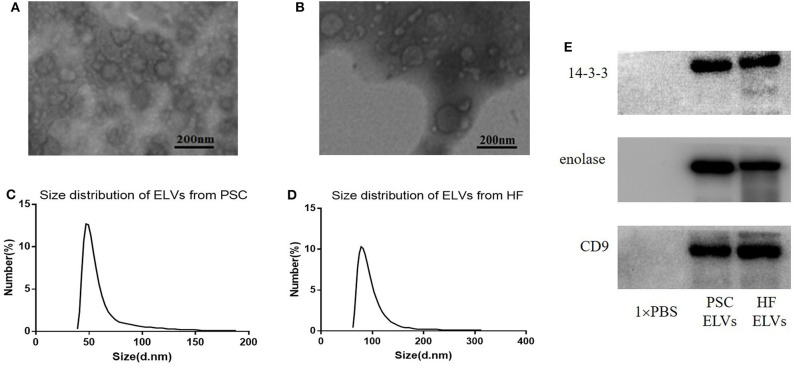
Identification ELVs from protoscoleces **(A,C)** and hydatid fluid **(B,D)**. **(A,B)** Images of the rounded or cup-shaped vesicles obtained using negative staining under TEM. Scale bars: 200 nm. **(C,D)** The diameter distribution analysis of the purified ELVs assessed using nanoparticle tracking analysis. **(E)** Exosome-associated markers 14-3-3, enolase, and CD9 were measured using Western blotting analysis.

Transmission electron microscopy and NTA were applied to evaluate the morphology and size distribution of the isolated PSC–ELVs and HF–ELVs. Spherical vesicles in the 30–150 nm range were observed under TEM (Figures 1A,B). Nanoparticle tracking analysis also showed that the majority of purified vesicles derived from PSCs and HF were between 40–70 nm and 60–90 nm in diameter, respectively (Figures 1C,D), which were compatible with the size of exosomes (Gillan et al., 2019). Western blotting analysis confirmed the presence of exosomal marker proteins, including 14-3-3, enolase, and CD9 (Figure 1E).

### Overview of Small RNAs Sequencing

Illumina Hiseq X Ten sequencing resulted in 23–27 million raw reads obtained per ELV. After trimming of adaptors and length filtering, high-quality (quality score, *Q* ≥ 20) clean reads were obtained from the two types of ELVs, with no significant differences in length distribution of small RNAs (21–23 nt) (Supplementary Figure 1), which were used for further analysis. BLASTN was used to search against the RFAM and GenBank databases using the pooled data for the two types of ELVs. Approximately 0.02–0.43% of the clean reads were annotated as rRNA, snRNA, snoRNA, or tRNA (Supplementary Table 2). The clean reads were compared with *E. granulosus* genome, and the results showed that 58.08–58.24% of the clean reads could map to *E. granulosus* (Supplementary Table 2).

### Identification of Conserved miRNAs in PSC–ELVs and HF–ELVs

The clean reads from the two types of ELVs were aligned to the *E. granulosus* database in miRBase for characterization. According to the database results, 118 miRNAs and 58 miRNAs were identified in PSC–ELVs and HF–ELVs, respectively. Among them, 72 and 48 miRNAs were known miRNAs belonging to *E. granulosus*, and the remaining 46 and 10 miRNAs were putative novel miRNAs identified by MiRDeep2. Among the 118 miRNAs in PSC–ELVs, 12 were very abundant (>10,000 reads) in the PSC–ELVs including egr-miR-4989-3p, egr-miR-125-5p, egr-let-7-5p, egr-miR-71-5p, egr-miR-61-3p, egr-miR-277a-3p, egr-miR-10a-5p, egr-bantam-3p, egr-miR-2162-3p, egr-miR-2a-3p, egr-let-7-3p, and egr-miR-96-5p (Supplementary Table 3). Intriguingly, among the 58 miRNAs in HF–ELVs, the most abundant miRNAs (counts >10,000 reads) were the same as those in the PSC–ELVs.

However, the expression levels of egr-miR-3479a-5p, egr-miR-1992-3p, egr-miR-3479b-5p, egr-miR-124a-5p, and some other miRNAs were rather low in PSC–ELVs. The expression levels of egr-miR-3479a-5p, egr-miR-8-3p, egr-miR-133-5p, egr-miR-7-3p, and some other miRNAs were rather low in HF–ELVs (Supplementary Table 3).

The 20 most abundant and known miRNAs identified from the sRNA library with read counts of more than 3,000 in PSC–ELVs are listed in Table 1. Egr-miR-4989-3p was the most abundant in the two types of ELVs, followed by egr-miR-125-5p and egr-let-7-5p in PSC–ELVs, and egr-let-7-5p and egr-miR-125-5p in HF–ELVs. Other miRNAs commonly found in ELVs derived from helminthes, such as miR-1, miR-9, and miR-10, were also found in the vesicles.

**Table 1:** Twenty known miRNAs abundant in PSC-ELVs and HF-ELVs.

**miRNA**	**HF-ELVs (read count)**	**PSC-ELVs (read count)**	**Sequence**	**Length**
egr-miR-4989-3p	731785	1218418	AAAATGCACCAACTATCTGAGA	22
egr-miR-125-5p	98624	214209	TCCCTGAGACCCTAGAGTTGTC	22
egr-let-7-5p	122110	186487	TGAGGTAGTGTTTCGAATGTCT	22
egr-miR-71-5p	78485	141374	TGAAAGACGATGGTAGTGAGA	21
egr-miR-61-3p	20891	102383	TGACTAGAAAGAGCACTCACATCC	24
egr-miR-277a-3p	14315	57284	TAAATGCATTTTCTGGCCCGTA	22
egr-miR-10a-5p	53478	45031	CACCCTGTAGACCCGAGTTTGA	22
egr-bantam-3p	27505	43375	TGAGATCGCGATTACAGCTGAT	22
egr-miR-2162-3p	12562	40877	TATTATGCAACTTTTCACTCC	21
egr-miR-2a-3p	19234	28392	AATCACAGCCCTGCTTGGAACC	22
egr-let-7-3p	10762	16581	ACATCCGTTTCACTATCTGCATA	23
egr-miR-96-5p	11001	14662	ATTGGCACTTTTGGAATTGTC	21
egr-miR-4991	9587	8628	GATCCTGGAATCCAACCTCATT	22
egr-miR-184-3p	7063	7938	GGGACGGAAGTCTGAAAGGTTT	22
egr-miR-7-5p	3843	7024	TGGAAGACTGGTGATATGTTGT	22
egr-miR-7b-5p	5685	6572	TGGAAGACTTGTGATTAGATTGTT	24
egr-miR-124b-3p	4012	6328	TAAGGCACGCGGTGAATACC	20
egr-miR-281-3p	3064	4389	TGTCATGGAGTTGCTCTCTATA	22
egr-miR-9-5p	1646	4173	TCTTTGGTTATCTAGCTGTGTG	22
egr-miR-3479a-3p	1395	3541	TATTGCACGTTCTTTCGCCATC	22

### Differentially Expressed miRNAs in PSC–ELVs and HF–ELVs

**Figure 2:**
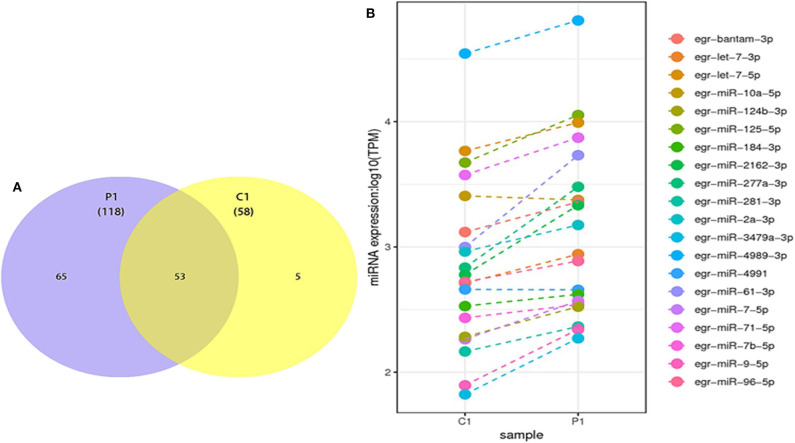
Discovery and profiling of miRNAs in PSC-ELVs and HF-ELVs. **(A)** Comparative numbers of miRNAs in PSC-ELVs and HF-ELVs. **(B)** Comparative expression analysis of the 20 most abundant miRNAs identified in PSC-ELVs and HF-ELVs. P1: PSC-ELVs. C1: HF-ELVs.

The differentially expressed miRNAs, including known and novel miRNAs between PSC–ELVs and HF–ELVs, were analyzed using high-throughput sequencing. Comparing the miRNAs numbers and expression levels of HF–ELVs with those of PSC–ELVs identified 53 miRNAs that were present in both types of ELVs, whereas 65 and 5 miRNAs uniquely existed in PSC–ELVs and HF–ELVs, respectively (Figure 2A). Eighty-five miRNAs were differentially expressed, among which 73 were upregulated and 12 were downregulated in PSC–ELVs (Supplementary Table 4). The differentially expressed miRNAs were defined as having a fold change ≥2.0 and *P* < 0.05. The expression levels of the 20 most abundant miRNAs in PSC–ELVs were higher than those of the corresponding miRNAs in HF–ELVs (Figure 2B). These results indicated that the miRNAs exhibited a wide range of expression levels in the two types of ELVs.

### qRT-PCR Validation

**Figure 3:**
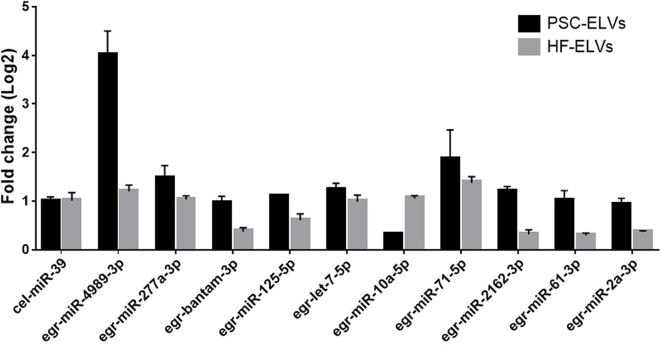
Identification of abundant miRNAs in PSC-ELVs and HF-ELVs using qPCR. Cel-miR-39 acted as external control to confirm normalization of sample-to-sample variation. Results are presented as log2-fold changes in expression ± standard error.

To verify the results of miRNAs sequencing, we used qRT-PCR to detect the expression of 10 abundant miRNAs selected from the both PSC–ELVs and HF–ELVs. The qRT-PCR results were consistent with the high-throughput sequencing, and these miRNAs were likely to play roles in response to the biological functions of ELVs in parasite development and infections (Figure 3).

### Identification of lncRNAs and circRNAs in PSC–ELVs and HF–ELVs

**Figure 4:**
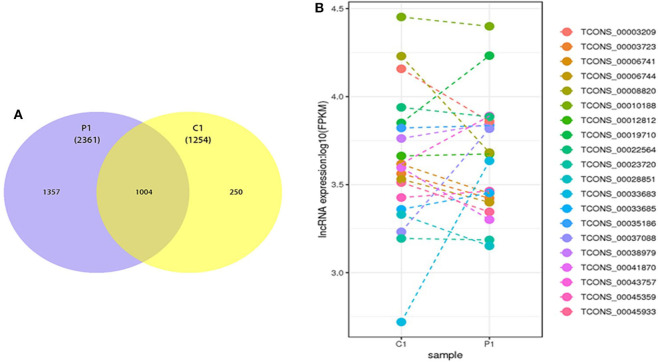
Discovery and profiling of lncRNAs in PSC-ELVs and HF-ELVs. **(A)** Comparative numbers of lncRNAs in PSC-ELVs and HF-ELVs. **(B)** Comparative expression analysis of the 20 most abundant lncRNAs identified in PSC-ELVs and HF-ELVs. P1: PSC-ELVs. C1: HF-ELVs.

According to the database results, 2,361 and 1,254 lncRNAs were identified in the two types of ELVs, respectively, among which 1,357 and 250 lncRNAs uniquely existed in PSC–ELVs and HF–ELVs, respectively, and 1,004 lncRNAs were present in both types of ELVs (Figure 4A). Among the 2,361 identified lncRNAs in PSC–ELVs, nine were the most abundant lncRNAs (counts >10,000) in the PSC–ELVs, including TCONS_00010188, TCONS_00043757, TCONS_00019710, TCONS_00038979, TCONS_00035186, TCONS_00003209, TCONS_00012812, TCONS_00006741, and TCONS_00003723 (Supplementary Table 5). Among the lncRNAs, 42 were differentially expressed, among which 19 were upregulated, and 23 were downregulated in PSC–ELVs. Intriguingly, among the 1,254 identified lncRNAs in HF–ELVs, the most abundant lncRNAs (counts >10,000) were almost the same as those in the PSC–ELVs.

However, the expression levels of TCONS_00006556, TCONS_00050975, TCONS_00050974, and some other lncRNAs were rather low in PSC–ELVs, whereas the expression levels of TCONS_00052899, TCONS_00018540, TCONS_00053812, and some other lncRNAs were rather low in HF–ELVs. Intriguingly, the expression levels of the 20 most abundant lncRNAs in PSC–ELVs were mostly lower than those of the corresponding lncRNAs in HF–ELVs (Figure 4B).

The clean reads were analyzed using the CIRI software for circRNAs prediction, which identified 1,277 and 512 circRNAs in PSC–ELVs and HF–ELVs, respectively. Among them, 1,094 and 329 known circRNAs existed uniquely in PSC–ELVs and HF–ELVs, respectively, and 183 circRNAs were present in both types of ELVs (Supplementary Figure 2A). Among the 1,277 identified circRNAs in PSC–ELVs, circRNA_1446, circRNA_0954, and circRNA_1451 were the most abundant circRNAs, whereas circRNA_1446, circRNA_0954, and circRNA_0977 were the most abundant circRNAs (counts >1,000 reads) in the HF–ELVs (Supplementary Table 6).

However, the expression levels of circRNA_0082, circRNA_0646, circRNA_1161, and some other circRNAs were rather low in PSC–ELVs, whereas circRNA_0082, circRNA_0646, circRNA_1162, and some other circRNAs were rather low in HF–ELVs (Supplementary Table 6). Meanwhile, the expression levels of the 20 most abundant circRNAs in PSC–ELVs were higher than those of the corresponding circRNAs in HF–ELVs, similar to the miRNAs (Supplementary Figure 2B).

### miRNA Target Prediction

Most studies focus on the mRNA targets of miRNAs, which can reduce protein expression either by inhibiting translation or by promoting target mRNA degradation. Furthermore, miRNAs play an important role in cross-species communication (Liang et al., 2013). The host target genes of the 20 most abundant miRNAs in PSC–ELVs were predicted using the miRanda software. In total, 895 target mRNAs were predicted against the 20 most abundant miRNAs (Supplementary Table 7). Among them, 13 miRNAs could bind to more than three genes, two miRNAs could bind to a single human mRNA, and five miRNAs had no predicted host target genes.

**Figure 5:**
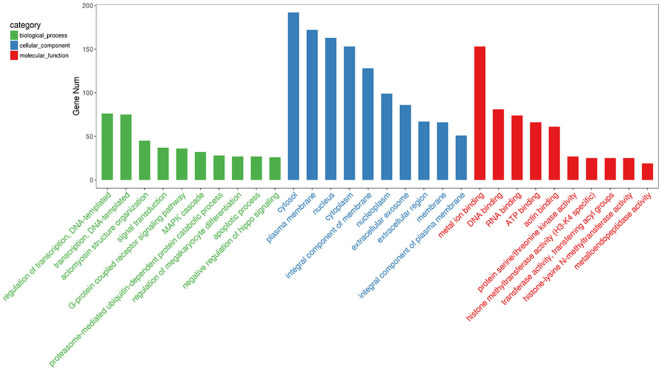
Top 30 GO terms from the genes enrichment analysis among the 20 most abundant miRNAs in the PSC-ELVs.

To gain insights into the functions of the 20 most abundant miRNAs in the PSC–ELVs, the host target genes were analyzed using the GO and KEGG databases. In the GO analysis, the host target genes of the 20 most abundant miRNAs in PSC–ELVs were mostly enriched in regulation of transcription, DNA-templated, and actomyosin structure organization in biological processes; cytoplasm, plasma membrane, and nucleus in cellular component; and metal ion binding, DNA binding, and RNA binding in molecular functions (Figure 5).

**Figure 6:**
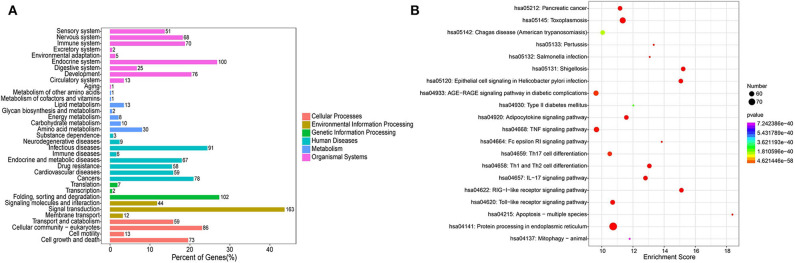
KEGG enrichment analysis of the predicted targets of the 20 most abundant miRNAs in the PSC-ELVs. **(A)** KEGG pathway classification. **(B)** Top 20 significant KEGG pathways.

The KEGG pathway analysis indicated 179 enriched pathways of human genes. The most significant enriched pathways were protein processing in endoplasmic reticulum, shigellosis, and RIG-I-like receptor signaling pathway for the PSC–ELVs miRNAs. The main KEGG pathway classification included signal transduction, folding, sorting, and degradation, endocrine system, and infectious diseases (Figure 6A). The miRNAs in parasite ELVs can be transferred to host cells to modulate host gene expression and the immune response. These most abundant miRNAs might also participate in inflammatory signaling pathways, such as the tumor necrosis factor (TNF) signaling pathway, the TLR signaling pathway, the mitogen-activated protein kinase (MAPK) signaling pathway, and the nuclear factor kappa B (NF-κB) signaling pathway. Additionally, KEGG analysis showed some pathways related to parasite infections, such as Th1 and Th2 cell differentiation pathway, Th17 cell differentiation pathway, and the IL-17 signaling pathway (Figure 6B). Therefore, these most abundant miRNAs in PSC–ELVs might regulate *E. granulosus* infectious and inflammatory processes.

### Interactions Among lncRNA-miRNA-mRNA

Many reports have indicated that RNA transcripts, such as mRNAs, lncRNAs, pseudogenes, and circRNAs, can act as competing endogenous RNAs (ceRNAs) or natural miRNA sponges, which communicate with and coregulate each other by competitive binding to shared miRNAs (Tay et al., 2014). Understanding the ceRNA interactions and crosstalk in intertwined networks will provide significant insights into gene regulation and will have implications in human disease. Therefore, the interaction between PSC–ELVs miRNAs and human mRNAs were analyzed using miRanda according to the miRNA–mRNA binding sites. Significantly, there were 23 miRNA–mRNA target pairs comprising 5 miRNAs and 23 mRNAs, which might play crucial roles in the host immune response and pathogenesis of *E. granulosus* infections. Three significant miRNAs, egr-miR-125-5p (degree = 11), egr-miR-10a-5p (degree = 7), and egr-miR-4991 (degree = 3) had the most target mRNAs. The host target genes of these miRNAs might be involved in the MAPK cascade (*NRG2, PSME2, MARK3*, and *PSMD6* genes), inflammatory response *(DDTL* and *ADORA2A* genes), collagen fibril organization and catabolic process (*COL5A2, COLGALT1, COL4A6*, and *ADAMTS14* genes), and apoptotic process (*NSMAF, UBE2D3, CRYAA2, TNFSF14, C19orf12, ITSN1, CRYAA*, and *ADORA2A* gene). Furthermore, egr-miR-125-5p and egr-miR-10a-5p share seed sequence (nucleotides 2–8) identity with *Homo sapiens* mature miRNAs (Supplementary Figure 3).

**Figure 7:**
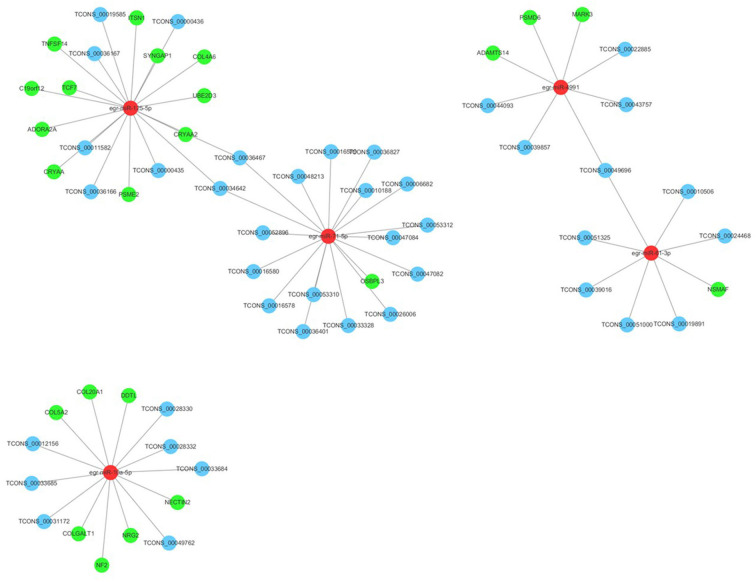
Long ncRNA–miRNA–mRNA interaction networks in PSC-ELVs. Red, light blue, and green circles represent miRNAs, lncRNAs, and mRNAs, respectively.

In addition, the lncRNAs regulated by miRNAs were analyzed using the miRanda algorithm. In total, 44 miRNA-lncRNA regulatory pairs were identified including 5 miRNAs and 41 lncRNAs. In the miRNA-lncRNA network, egr-miR-71-5p (degree = 17), egr-miR-125-5p (degree = 8), egr-miR-10a-5p (degree = 6), and egr-miR-61-3p (degree = 6) had the most target lncRNAs. Based on the regulatory pairs of miRNA–mRNA and miRNA-lncRNA, a ceRNA network consisting of 5 miRNAs, 41 lncRNAs, and 23 mRNAs was constructed (Figure 7). Each mRNA or lncRNA could be regulated by one or more miRNAs and *vice versa*. The constructed ceRNA network was conducive to depicting the role of miRNAs during *E. granulosus* infection.

## Discussion

A true exchange of biological macromolecules across of the laminated layer occurs between the parasite and the host, with constant vesicular trafficking through the tegument (Diaz et al., 2011; Teichmann et al., 2015). *Echinococcus granulosus* ELVs were observed in the metacestode culture medium, possibly because extracellular InsP6 (Inositol hexaphosphoric acid) binds to proteins on *E. granulosus* ELVs, acting as a “dynamic anchorage” that promotes their passage across the laminated layer (Nicolao et al., 2019). Recent studies also confirmed that parasite-derived ELVs participate in intercellular communication, immune responses, and pathology, in which a number of ncRNAs in ELVs play a crucial role during these processes (Buck et al., 2014; Hansen et al., 2019). Integration analysis of ncRNAs in parasite-derived ELVs would help understand the parasite–host interactions. Nevertheless, it is unknown whether *E. granulosus–*derived ELVs contain ncRNAs and whether ELVs miRNAs could target host genes to modulate the immune responses. In the present study, we isolated and characterized the ELVs produced from *in vitro* cultures of *E. granulosus* PSCs and HF obtained from naturally infected sheep. The isolated PSC–ELVs and HF–ELVs were similar in size and morphology to the ELVs characterized in previous reports that referred to *E. granulosus* PSCs, metacestodes, fertile sheep hydatid cysts, and other helminths (*Echinostoma caproni, F. hepatica, S. japonicum, Brugia malayi*, and *Heligmosomoides polygyrus*) (Marcilla et al., 2012; Buck et al., 2014; Zamanian et al., 2015; Zhu et al., 2016a).

Larval PSCs are an important transition of the life cycle stage, capable of developing either into a secondary hydatid cyst in the intermediate host or an adult worm in the final host; therefore, we compared the numbers and expression levels of ncRNAs in PSC–ELVs with those in HF–ELVs to identify the miRNAs and lncRNAs that might be crucial in the host immune response. There were 53 miRNAs and 1,130 lncRNAs that were present in both types of ELVs. In addition, there were more miRNAs and lncRNAs in the PSC–ELVs than in the HF–ELVs; however, the 20 most abundant miRNAs were almost the same in both types of ELVs. Intriguingly, the expression levels of the 20 most abundant miRNAs and circRNAs in PSC–ELVs were higher than those of the corresponding miRNAs and circRNAs in HF–ELVs, whereas the expression levels of the 20 most abundant lncRNAs in PSC–ELVs were mostly lower than those of corresponding lncRNAs in HF–ELVs. *Echinococcus granulosus* cysts (in particular PSCs and the GL) produce and release HF–ELVs; therefore, HF–ELVs and PSC–ELVs have certain common biological components such as ncRNAs; however, their numbers and expression levels are different. Given that PSCs spilled from the cysts can develop into secondary cysts, which is the most frequent reason for relapse, we propose that PSCs spilled from the cysts are more active in excreting ELVs containing ncRNAs. Cucher et al. (2011) identified 38 miRNAs from *E. granulosus* and found that miR-2, miR-71, miR-9, miR-10, let-7, and miR-277 were enriched in PSCs and cyst walls of secondary hydatid cysts, in which their expression in different life cycle stages might be related to parasite development. These miRNAs were also identified in PSC–ELVs and HF–ELVs, which indicated that these miRNAs of PSCs and hydatid cysts could be encapsulated into their excretory ELVs. Moreover, another study compared the expression levels of miRNAs in adult worms or the cyst membrane with those in the PSCs and identified miR-125, miR-277, and miR-4989 upregulated in adult worms but downregulated in the cyst membrane (Bai et al., 2014). Let-7 has been shown to be positively correlated with its putative target gene (vitamin D receptor), which may promote the PSCs to develop into adult worms (Bai et al., 2014). In our study, compared with the ncRNAs in HF–ELVs, there were 85 miRNAs and 42 lncRNAs that were differentially expressed, among which 73 miRNAs and 19 lncRNAs were upregulated, and 12 miRNAs and 23 lncRNAs were downregulated in PSC–ELVs. miR-125-5p, miR-277a-3p, and miR-4989-5p were upregulated in PSC–ELVs, but downregulated in HF–ELVs. miR-4989 is the most abundant miRNA in both ELVs and belongs to the miR-277 family. Recently, miR-4989 and miR-277 have been reported to regulate the transcriptional landscape during juvenile to adult transition in *Schistosoma mansoni* (Protasio et al., 2017). miR-4989 was confirmed to be encapsulated into *Echinococcus multilocularis–*derived ELVs and was demonstrated to be capable of modulating nitric oxide production, the expression of TNF-α, and the key components in the LPS/TLR4 signaling pathway in RAW264.7 macrophages (Ding et al., 2019). Furthermore, *E. multilocularis* miR-4989-3p and miR-277 were detected in the sera of infected mice (Guo and Zheng, 2017). These results simply that the differential expression levels of ncRNAs in the two types of ELVs from different life stages might be involved in parasite development, and some miRNAs might moderate the host immune response.

Parasite-derived miRNAs could be involved in host immunity and pathogenesis via cross-species regulation of host mRNAs (Hu et al., 2019; He et al., 2020; Wang et al., 2020). Previous reports have shown that *S. japonicum*–derived miR-2162 can directly promote host hepatic fibrosis through cross-species regulation of host transforming growth factor β receptor III (He et al., 2020), and this miRNA was present in *S. japonicum* egg ELVs (Zhu et al., 2016b). For further analysis of the biological functions of *E. granulosus* ELVs in the parasite–host interaction, the 20 most abundant miRNAs in PSC–ELVs were selected to predict their host target genes. The GO enrichment analysis showed that these target mRNAs, which were inversely correlated with miRNAs, were involved in signal transduction, biological regulation, immune system processes, and other cellular processes. KEGG pathway analysis further demonstrated that the mRNAs targeted by the miRNAs were mainly involved in the regulation of signal transduction, infectious diseases, and the immune system, which have been widely researched and demonstrated to be associated with the functions of exosomes (Coakley et al., 2015). The predicted host target genes are involved in the inflammatory responses, including encoding members of the TNF, TLR, MAPK, and NF-κB signaling pathways, which have previously been documented to modulate cytokines during chronic *E. granulosus* infection (Refik et al., 2005; Tuxun et al., 2015; Labsi et al., 2018; Zhang et al., 2019). Additionally, the most abundant miRNAs were predicted to participate in Th1 and Th2 cell differentiation, Th17 cell differentiation, and IL-17 signaling pathway, and these immune responses occur during *E. granulosus* infections (Tuxun et al., 2012; Labsi et al., 2018). These results indicated that these 20 most abundant miRNAs likely participate in parasite–host interactions and might regulate the host immune response. A better understanding of the immune response induced by *E. granulosus* will help to further clarify the parasite–host interaction and provide a scientific basis for its treatment.

To further study the roles of PSC–ELVs miRNAs in immunity and pathogenicity, the specific miRNA sequences were compared with the corresponding host miRNAs, and an lncRNA–miRNA–mRNA regulatory network was constructed to investigate their correlations. The integrative analysis of the ceRNA interaction network demonstrated the regulatory functions of miRNAs and the specific interplay with other RNAs via the lncRNA–miRNA–mRNA regulatory axis during the parasite–host interactions. In the ceRNA network, five core miRNAs (egr-miR-125-5p, egr-miR-71-5p, egr-miR-4991, egr-miR-61-3p, and egr-miR-10a-5p) and 41 core lncRNAs were identified according to the literature and the regulatory relationship between lncRNAs and miRNAs. Intriguingly, three core lncRNAs (TCONS_00010188, TCONS_00043757, and TCONS_00033685) were also among the 20 most abundant lncRNAs in PSC–ELVs, which indicated that there may be some correlations between the most abundant miRNAs and lncRNAs in the PSC–ELVs. For the miRNAs, miR-61 showed significantly higher expression in PSCs under exposure to long-term or high albendazole sulfoxide drug levels, indicating that miR-61 might be a potential new biomarker to assess the response to chemotherapy (Mortezaei et al., 2019). In the present study, egr-miR-125-5p was predicted to target the *CRYAA, ADORA2A, COL4A6*, and *PSME2* genes, which were involved in apoptosis, inflammatory response, collagen catabolic process, and MAPK cascade, respectively (Sund et al., 2005; Billing et al., 2011; Li et al., 2017; Kobold et al., 2019). miR-125b has been shown to regulate inflammatory responses by desensitizing TLR activation after recognition of pathogens (Gracias and Katsikis, 2011). In addition, miR-125 promoted apoptosis by reducing the expression of *VEGF* (Wu et al., 2018), which is possibly associated with the neovascularization required by the hydatid cyst metabolism in patients with cystic echinococcosis (Matera et al., 2018). Egr-miR-10a-5p was predicted to target the *NECTIN2, COLGALT1, COL5A2*, and *DDTL* genes, which were involved in the positive regulation of natural killer cell–mediated cytotoxicity and T-cell receptor signaling pathway, collagen fibril organization, collagen catabolic process, tumor necrosis factor production, and ERK1 and ERK2 cascades, respectively (Stanietsky et al., 2013; Park et al., 2017; Miyatake et al., 2018). It has been reported that hsa-miR-10a-5p had a role in regulating the proliferation and invasiveness of cancer cells and inflammatory responses in endothelial cells (Vaher et al., 2019). miR-10a-5p inhibited *MAP3K7* and β-transducin repeat-containing gene expression, thereby reducing NF-κB activation and displaying anti-inflammatory effects in the athero-susceptible endothelium (Fang et al., 2010). Further study showed that PSC–ELVs could be internalized by dendritic cells and induce their maturation (Nicolao et al., 2019). These abundant miRNAs were detected in dendritic cells upon internalization of PSC–ELVs and induced dendritic cells to secrete certain inflammatory cytokines (unpublished data). The ceRNAs predicted in the present work provided a theoretical basis for further study of how *E. granulosus* ELVs miRNAs moderate the host immune response. More importantly, egr-miR-125-5p and egr-miR-10a-5p shared seed site sequence identity with *H. sapiens* mature miRNAs and might alter the corresponding host miRNA expression, representing a potential advantage for parasite invasion. Therefore, it was reasonable to propose that these miRNAs in PSC–ELVs participate in parasitic infections, immune responses, and pathogenesis by acting in concert with their correlated mRNAs and lncRNAs.

## Conclusions

Exosome-like vesicles are critical for intercellular communication, modulation of immune responses, and pathology. This is the first study to analyze the ncRNAs (miRNAs, circRNAs, and lncRNAs) profiles of the PSC–ELVs and HF–ELVs. In addition, a lncRNA–miRNA–mRNA regulatory network was constructed according to the regulatory mechanisms of miRNAs, which provided new ideas and a theoretical basis for further clarification of the function and mechanism of *E. granulosus* ELVs miRNAs in the parasite–host interaction. Egr-miR-125-5p and egr-miR-10a-5p, sharing identical seed sites with host miRNAs, were predicted to mediate the immune response during parasite infections. Although these abundant miRNAs might be involved in the host immune response and pathogenesis, the sensitivity and specificity of functional miRNAs as potential biomarkers or novel treatment strategies for cystic echinococcosis should be further investigated. Understanding the underlying mechanisms and functions of the ncRNAs in ELVs will pave the way for new parasite vaccine strategies, diagnostic markers, and treatments.

## Data Availability Statement

The miRNA and lncRNA datasets generated for this study can be found in the BioProject under accession numbers PRJNA573515 and PRJNA592079, respectively. (https://www.ncbi.nlm.nih.gov/bioproject/PRJNA573515, https://www.ncbi.nlm.nih.gov/bioproject/PRJNA592079).

## Author Contributions

YS and JC conceived and designed the study. XZ, WG, SC, JY, and JZ performed the experiments and data analysis. YS and JC contributed reagents and materials. XZ wrote the manuscript. YS and JC revised the manuscript. All authors read and approved the final version of the manuscript.

## Conflict of Interest

The authors declare that the research was conducted in the absence of any commercial or financial relationships that could be construed as a potential conflict of interest.

## Supplementary Material

Supplementary Figure 1 Length distribution of small RNAs in ELVs derived from PSCs and HF. P1: PSC-ELVs. C1: HF-ELVs.

Supplementary Figure 2 Discovery and profiling of circRNAs in PSC-ELVs and HF-ELVs. (JPG 337 kb). **(A)** Comparative numbers of circRNAs in PSC-ELVs and HF-ELVs. **(B)** Comparative expression analysis of the 20 most abundant circRNAs identified in PSC-ELVs and HF-ELVs. P1: PSC-ELVs. C1: HF-ELVs.

Supplementary Figure 3 PSC-ELVs and HF-ELVs miRNA sequence homology to Homo sapiens miRNAs. (JPG 432 kb). miRNAs from PSC-ELVs, HF-ELVs, and *Homo sapiens* were grouped by seed site sequence identity (nucleotides 2–8) for the sequence alignments.

Supplementary Table 1 Primers used for qPCR detection of selected miRNAs. (XLSX 19 kb).

Supplementary Table 2 Distribution of small RNAs among different categories in two samples. (DOCX 15 kb).

Supplementary Table 3 The numbers and expression levels of miRNAs in PSC-ELVs and HF-ELVs. (XLSX 40 kb).

Supplementary Table 4 Differential expression profiles of miRNAs. (XLSX 34 kb).

Supplementary Table 5 The numbers and expression levels of lncRNAs in PSC-ELVs and HF-ELVs. (XLSX 676 kb).

Supplementary Table 6 The numbers and expression levels of circRNAs in PSC-ELVs and HF-ELVs. (XLSX 303 kb).

Supplementary Table 7 The host target genes of the 20 most abundant miRNAs in PSC-ELVs. (XLSX 280 kb).
