# Supplementary figures and images for: Comprehensive Analysis of Non-coding RNA Profiles of Exosome-Like Vesicles From the Protoscoleces and Hydatid Cyst Fluid of Echinococcus granulosus
Source: Front Cell Infect Microbiol. 2020 Jul 22;10:316. doi: 10.3389/fcimb.2020.00316 (PMC7387405; doi:10.3389/fcimb.2020.00316)

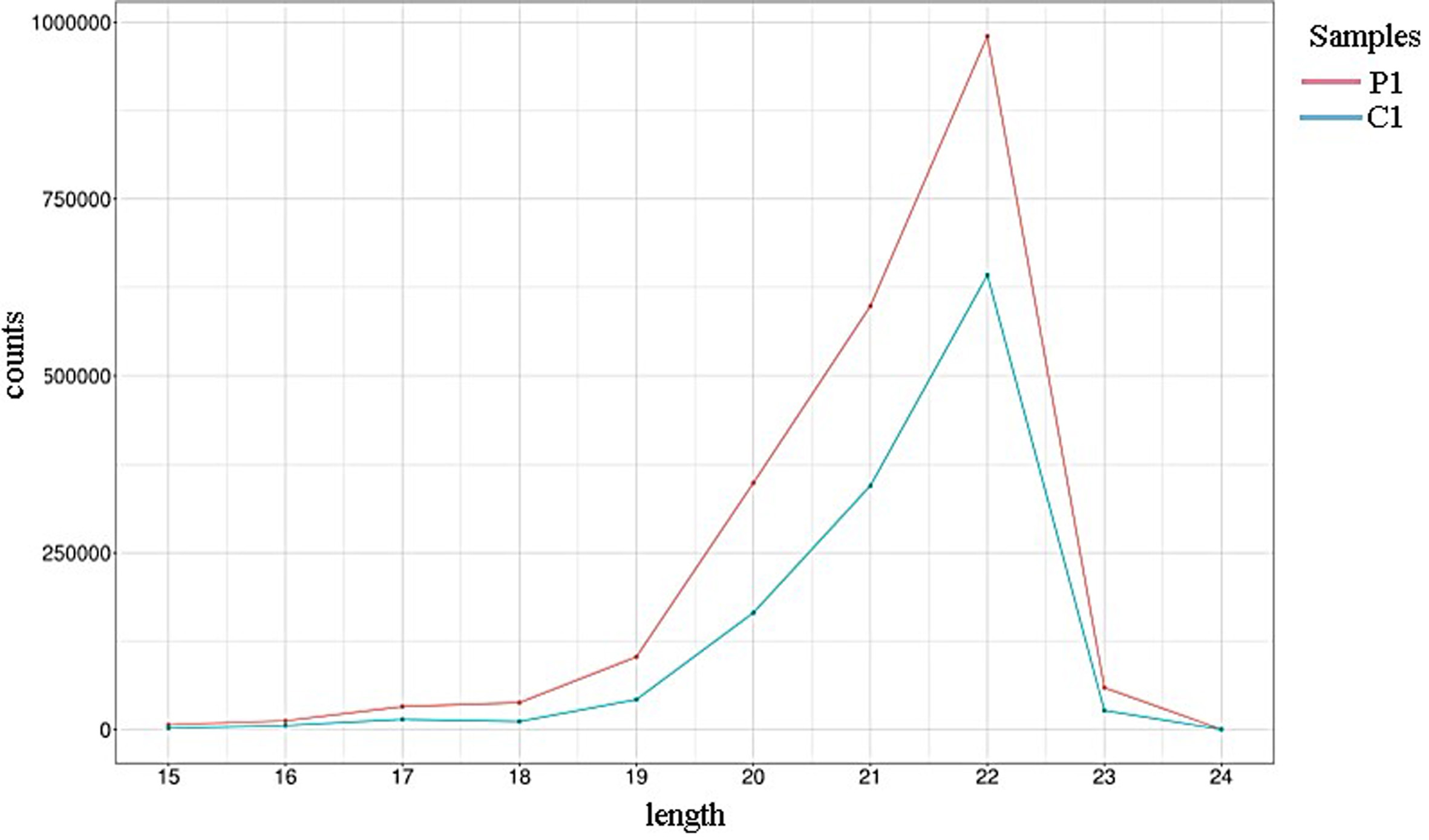

Supplement: Supplementary Figure 1 — Length distribution of small RNAs in ELVs derived from PSCs and HF. P1: PSC-ELVs. C1: HF-ELVs. [file Image_1.JPEG]

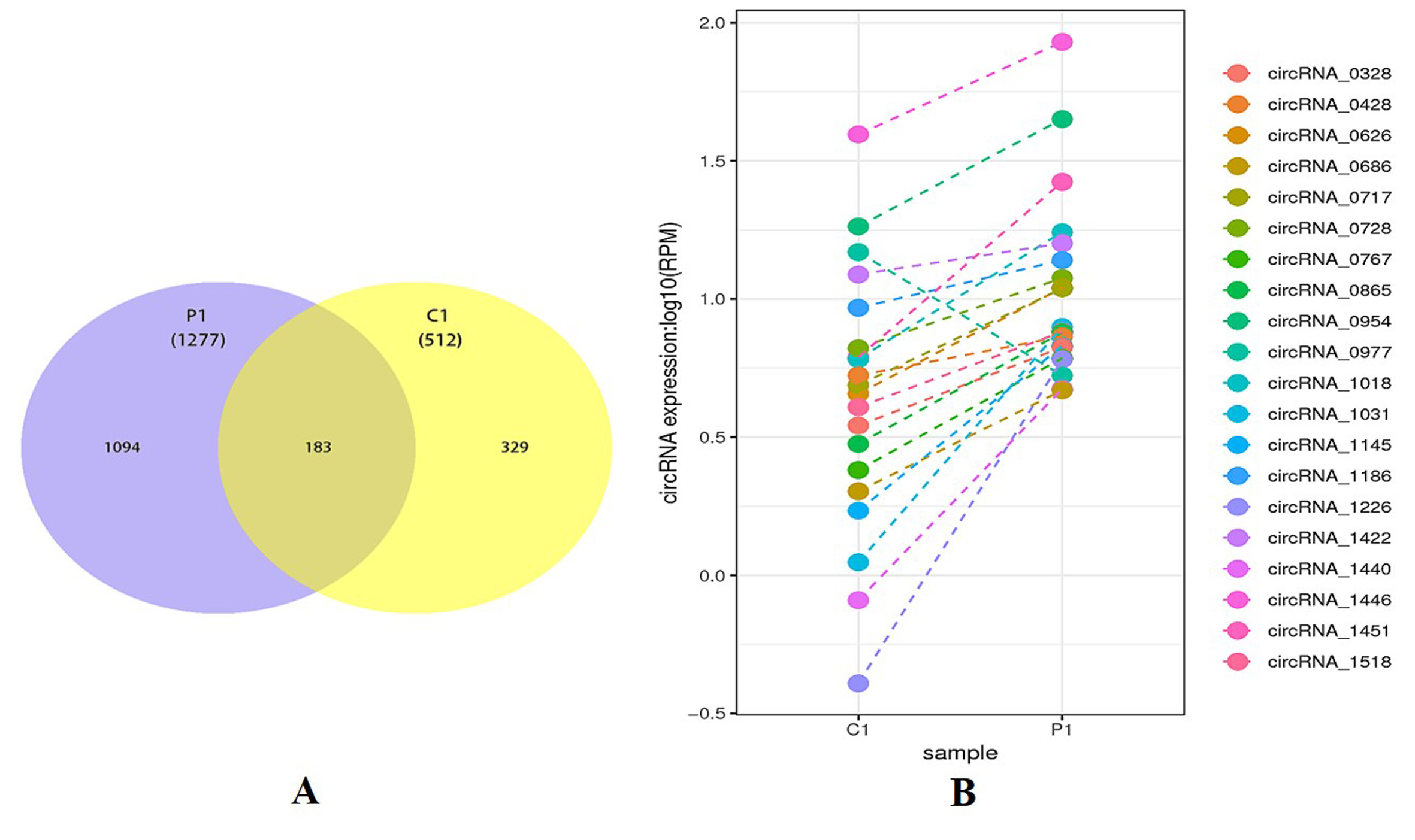

Supplement: Supplementary Figure 2 — Discovery and profiling of circRNAs in PSC-ELVs and HF-ELVs. (JPG 337 kb). (A) Comparative numbers of circRNAs in PSC-ELVs and HF-ELVs. (B) Comparative expression analysis of the 20 most abundant circRNAs identified in PSC-ELVs and HF-ELVs. P1: PSC-ELVs. C1: HF-ELVs. [file Image_2.JPEG]

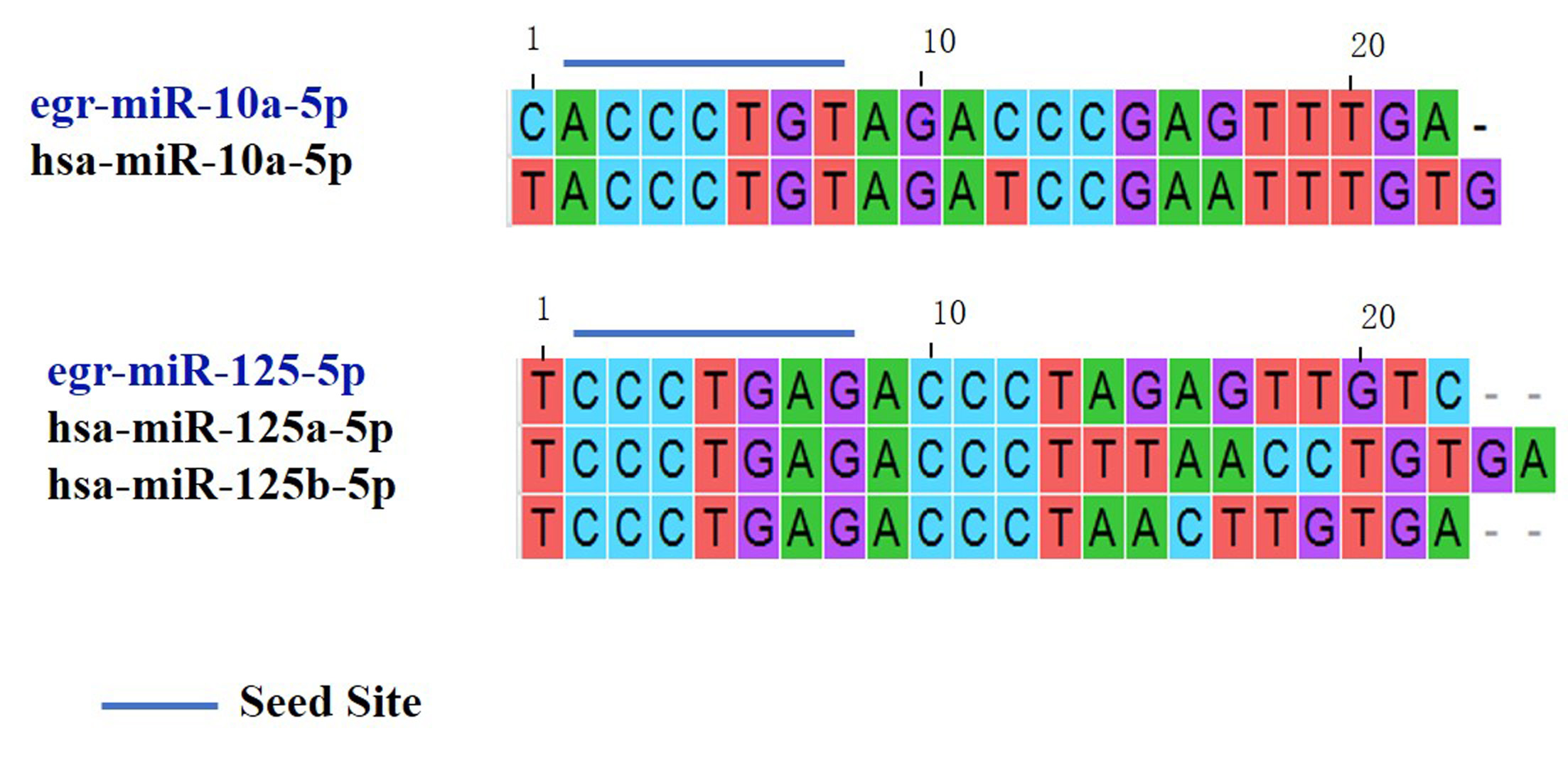

Supplement: Supplementary Figure 3 — PSC-ELVs and HF-ELVs miRNA sequence homology to Homo sapiens miRNAs. (JPG 432 kb). miRNAs from PSC-ELVs, HF-ELVs, and Homo sapiens were grouped by seed site sequence identity (nucleotides 2–8) for the sequence alignments. [file Image_3.JPEG]
