# Supplementary material for: Comprehensive Analysis of Non-coding RNA Profiles of Exosome-Like Vesicles From the Protoscoleces and Hydatid Cyst Fluid of Echinococcus granulosus
Source: Front Cell Infect Microbiol. 2020 Jul 22;10:316. doi: 10.3389/fcimb.2020.00316 (PMC7387405; doi:10.3389/fcimb.2020.00316)
Supplement: Supplementary Table 2 — Distribution of small RNAs among different categories in two samples. (DOCX 15 kb). [file Table_2.docx]

**Supplementary Table 2** Distribution of small RNAs among different categories in two samples

| **Sample** | **rRNA** | **tRNA** | **snRNA** | **Cis-reg** | **others** | **Aligned_reads^a^** | **Aligned^a^ (%)** |
| --- | --- | --- | --- | --- | --- | --- | --- |
| **PSC-ELVs** | 25292 | 3714 | 40172 | 29424 | 82058 | 11051635 | 58.24 |
| **HF-ELVs** | 18919 | 4687 | 37449 | 23890 | 69026 | 12160309 | 58.08 |

^a^ The unannotated clean reads were aligned with the genome of *Echinococcus granulosus*.

Aligned rate (%) = Aligned reads/clean reads × 100.
